# Pentadecanoylcarnitine is a newly discovered endocannabinoid with pleiotropic activities relevant to supporting physical and mental health

**DOI:** 10.1038/s41598-022-18266-w

**Published:** 2022-08-23

**Authors:** Stephanie Venn-Watson, John Reiner, Eric D. Jensen

**Affiliations:** 1 Epitracker Inc., San Diego, CA 92106 USA; 2 Seraphina Therapeutics, Inc., San Diego, CA 92106 USA; 3 US Navy Marine Mammal Program, Naval Warfare Information Center Pacific, San Diego, CA 92106 USA

**Keywords:** Molecular biology, Molecular medicine

## Abstract

As an emerging dietary essential fatty acid, pentadecanoic acid (C15:0) is expected to have bioactive metabolites with broad health benefits. Here, we evaluated pentadecanoylcarnitine, an endogenous C15:0 metabolite, for dose dependent cell-based activities, including measurement of its effects on 148 clinically relevant biomarkers across twelve primary human cell systems mimicking various disease states. Mechanisms of action for pentadecanoylcarnitine were also assessed across 78 cell-based target assays. Pentadecanoylcarnitine had dose-dependent anti-inflammatory activities, including lower IL-1α, ITAC, MCP-1, and IP-10, across five cell systems relevant to treating cardiovascular, immune, neoplastic, pulmonary, and skin diseases. Targeted assays showed pentadecanoylcarnitine as a full-acting cannabinoid 1 and 2 receptor agonist (EC50 3.7 and 3.2 µM, 111% and 106% maximum activity compared to the positive control, respectively). Pentadecanoylcarnitine also had 5-HT1A and 5-HT1B receptor agonist and histamine H1 and H2 receptor antagonist activities. In summary, pentadecanoylcarnitine, a second discovered full-acting endocannabinoid, had broad pleiotropic activities relevant to regulating inflammation, pain, mood, and sleep. This study’s findings further the need to evaluate the potential health impacts of C15:0 nutritional deficiencies caused by population-wide avoidance of all dietary saturated fats, including C15:0.

## Introduction

Pentadecanoic acid (C15:0), an odd-chain saturated fatty acid present in trace levels in dairy fat, as well as some types of fish and plants, is emerging as an essential fatty acid needed in the diet to maintain physiological health^1,2^. Large, prospective cohort studies have linked higher circulating concentrations of C15:0 to lower risks of developing chronic diseases, including cardiovascular disease, heart failure, and type 2 diabetes^3–5^. Further, higher dietary intake and circulating concentrations of C15:0 have been associated with lower mortality and greater longevity^5–7^.

Beyond association, C15:0 has been shown to be an active and beneficial nutrient. Daily oral C15:0 supplementation over 12 weeks effectively lowered glucose, cholesterol, pro-inflammatory cytokines, and body weight gain in a model of obesity and type 2 diabetes^2^. C15:0 supplementation also lowered inflammation, cholesterol, and triglycerides and attenuated anemia and liver fibrosis in a model of nonalcoholic fatty liver disease^2^. In human cell-based systems mimicking various disease states, C15:0 had dose-dependent anti-inflammatory and antifibrotic properties^2^. C15:0’s broad health benefits have been attributed to several mechanisms of action, including its role as a peroxisome proliferator-activated receptor α/δ agonist, an AMP-activated protein kinase activator, and a histone deacetylase 6 inhibitor, as well as its ability to repair mitochondrial function, improve the stability of red blood cell membranes, and decrease proliferation of cancer cells^8–11^.

Currently, there are two well-established essential fatty acids, alpha-linolenic acid (an omega-3 fatty acid) and linoleic acid (an omega-6 fatty acid)^12^. These dietary polyunsaturated fatty acids are critical precursors to endogenously produced metabolites, including eicosapentaenoic acid, docosahexaenoic acid, and arachidonic acid. In turn, arachidonic acid is used to produce prostaglandins and leukotrienes that affect inflammation, as well as anandamide and 2-arachidonoylglycerol, two primary endocannabinoids within the endocannabinoid system that regulate inflammation, pain, appetite, mood, and sleep^13–15^. Thus, essential fatty acids are expected not only to provide direct benefits, but to serve as precursors to downstream metabolites that are endogenously produced and play distinct roles in maintaining physiological health.

Similar to humans, bottlenose dolphins (*Tursiops truncatus*) are long-lived, large-brained mammals with demonstrated associations between higher circulating C15:0 and C17:0 concentrations and lower risks of chronic metabolic conditions^11,16^. When dolphins were fed a modified, high-C15:0 fish diet, their serum metabolome shifted, resulting in lower insulin and cholesterol, as well as attenuated anemia^11,16^.

To evaluate biological activities of key C15:0 metabolites, we leveraged dolphin serum metabolomic data to identify high-priority C15:0 metabolites from dolphins fed a C15:0-rich fish diet. With the identification of pentadecanoylcarnitine, we tested this endogenous C15:0 acylcarnitine metabolite for clinically relevant efficacies and activities.

## Results

### Pentadecanoylcarnitine is the highest-ranking metabolite resulting from a modified, higher-C15:0 fish diet

**Figure 1:**
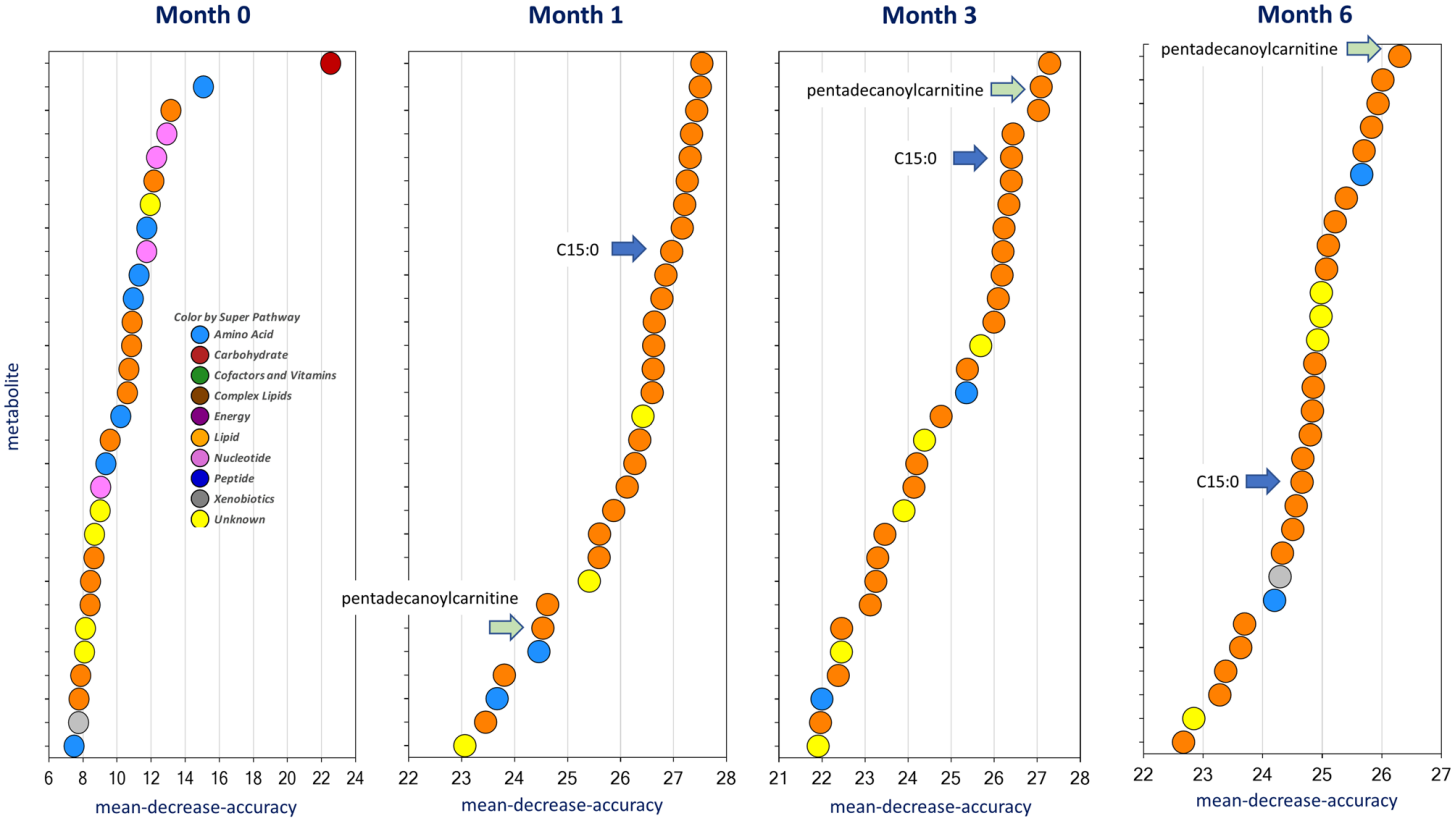
Top 30 biochemicals predicting dolphins on a modified, higher-C15:0 fish diet. Random Forest biochemical importance plot of the top 30 biochemicals in the bottlenose dolphin (*Tursiops truncatus*) serum metabolome that predicted dolphins on modified fish diet compared to dolphins on the baseline diet for Months 0 (baseline), 1, 3, and 6. Rankings of C15:0 and pentadecanoylcarnitine, by Month, are pointed out.

**Figure 2:**
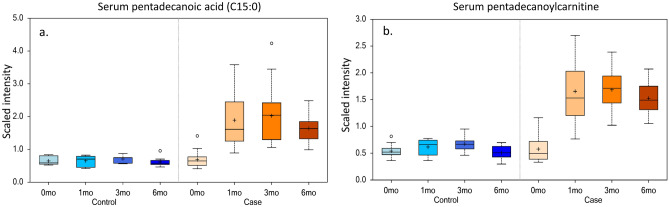
Increased serum C15:0 and pentadecanoylcarnitine concentrations among dolphins on a modified, higher-C15:0 fish diet. Relative changes in serum concentrations of (**a**) pentadecanoic acid (C15:0) and (**b**) pentadecanoylcarnitine during Months 0, 1, 3 and 6 among 10 bottlenose dolphins (*Tursiops truncatus*) fed a baseline fish diet (Control) and 20 dolphins fed a modified fish diet with higher C15:0 (Case).

As previously shared, a total of 819 small molecules were measured in the dolphin serum metabolome, and 241 were characterized as changed (*p* ≤ 0.05) due to the main effect of the modified fish diet^11^. Using hierarchical clustering and random forest regression, the top 30 prioritized compounds that were changed by the diet were 100% predictive of dolphins on the modified fish diet by Month 1. While neither C15:0 nor C15:0 metabolites were ranked among the top 30 compounds at Month 0, C15:0 ranked as the 9th, 5th, and 19th most important compound at Months 1, 3 and 6, respectively (Fig. 1). Only one clear C15:0 metabolite, pentadecanoylcarnitine, was present among the top 30 biochemicals, starting at Month 1. Pentadecanoylcarnitine was not ranked at Month 0, but it ranked as the 25th, 2nd, and 1st most important biochemical at Months 1, 3 and 6, respectively (Fig. 1). The reversal in rankings between C15:0 and pentadecanoylcarnitine over time was independent of similar relative increases in serum concentrations of both compounds over time (Fig. 2).

### Pentadecanoylcarnitine has broad dose-dependent antiproliferative and anti-inflammatory activities

As the top predictive compound from the dolphins’ modified diet study, which was also a metabolite containing C15:0, pentadecanoylcarnitine was tested for direct cell-based effects, including 148 clinically relevant biomarker readouts across 12 primary human cell-based systems mimicking various disease states.

**Figure 3:**
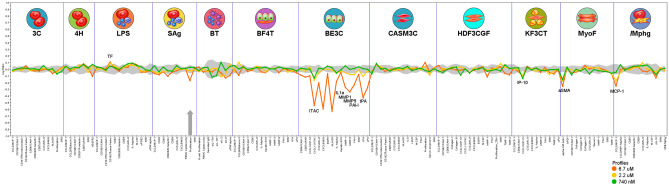
Clinically relevant activities of pentadecanoylcarnitine among 12 primary human cell systems mimicking various disease states. The X-axis lists the quantitative protein-based biomarker readouts measured in each system. The Y-axis represents a log-transformed ratio of the biomarker readouts for the drug-treated sample (n = 1) over vehicle controls (n ≥ 6). The grey region around the Y-axis represents the 95% significance envelope generated from historical vehicle controls. Biomarker activities are annotated when 2 or more consecutive concentrations change in the same direction relative to vehicle controls, are outside of the significance envelope, and have at least one concentration with an effect size > 20% (|log_10_ *ratio*|> 0.1). Antiproliferative effects are indicated by a thick grey arrow.

Here, pentadecanoylcarnitine had annotated, dose-dependent activities in 5 (42%) of the 12 systems (Fig. 3, Table 1). Pentadecanoylcarnitine had antiproliferative effects on T cells in the SAg system mimicking chronic inflammation, autoimmune disease, and hematological oncology (Fig. 3). At concentrations ranging between 0.7 and 6.7 µM, pentadecanoylcarnitine had dose-dependent anti-inflammatory activities, including lowered interleukin 1 alpha (IL-1α), interferon-inducible T-cell alpha chemoattractant (ITAC), monocyte chemoattractant protein 1 (MCP-1), and interferon-inducible protein 10 (IP-10), across three additional cell systems relevant to lung disease, dermatitis, chronic inflammation, and cardiovascular disease (Fig. 3). Pentadecanoylcarnitine also had dose-dependent tissue remodeling activities in two cell systems relevant to lung disease, fibrosis, and wound healing, including lowered plasminogen activation inhibitor 1 (PAI-1), matrix metallopeptidase 1 and 9 (MMP1, MMP9), and tissue plasminogen activator (tPA).

**Table 1:** Annotated, clinically relevant, and dose-dependent activities of pentadecanoylcarnitine among 4 primary human cell systems mimicking various disease states.

BioMAP cell system	Disease relevance	Annotated biomarker	Log-transformed ratio of the biomarker readouts for the treated sample over vehicle controls		
			0.7 µM	2.2 µM	6.7 µM
SAg	Chronic inflammation, autoimmune disease	T cell proliferation	0.03	− 0.03	− 0.17*^+^
BE3C	Chronic obstructive pulmonary disease, lung inflammation	ITAC	− 0.13*	− 0.17*^+^	− 0.54*^+^
		IL-1a	− 0.06*	− 0.06*	− 0.19*^+^
		MMP-1	0.05	− 0.05*	− 0.27*^+^
		MMP-9	0.00	− 0.08*	− 0.34*^+^
		PAI-1	0.01	− 0.08*	− 0.24*^+^
		tPA	0.01	− 0.11*	− 0.43*^+^
KF3CT	Dermatitis, psoriasis	IP-10	− 0.14*^+^	− 0.09*	− 0.10*^+^
MyoF	Wound healing, matrix remodeling, fibrosis, chronic inflammation	alpha-SM actin	− 0.16*^+^	− 0.18*^+^	− 0.26*^+^
Mphg	Chronic inflammation, cardiovascular disease	MCP-1	− 0.05	− 0.17*^+^	− 0.25*^+^

*Outside of the 95% significance envelope from controls. ^+^Effect size > 20% (|log10 *ratio*|> 0.1).

Biomarker activities are annotated when 2 or more consecutive concentrations change in the same direction relative to vehicle controls, are outside of the significance envelope, and have at least one concentration with an effect size > 20% (|log10 *ratio*|> 0.1).

### Pentadecanoylcarnitine is a full-acting endocannabinoid with pleiotropic activities relevant to both physical and mental health

Following the demonstration of clinically relevant activities in human cell systems, pentadecanoylcarnitine was assessed for dose–response agonist and antagonist activities by testing 10 doses across 78 assays and 47 genes and targets that are relevant to pharmacological mechanisms of action, including G-protein coupled receptors, kinases, transporters, ion channels, nuclear receptors, and non-kinase enzymes.

**Figure 4:**
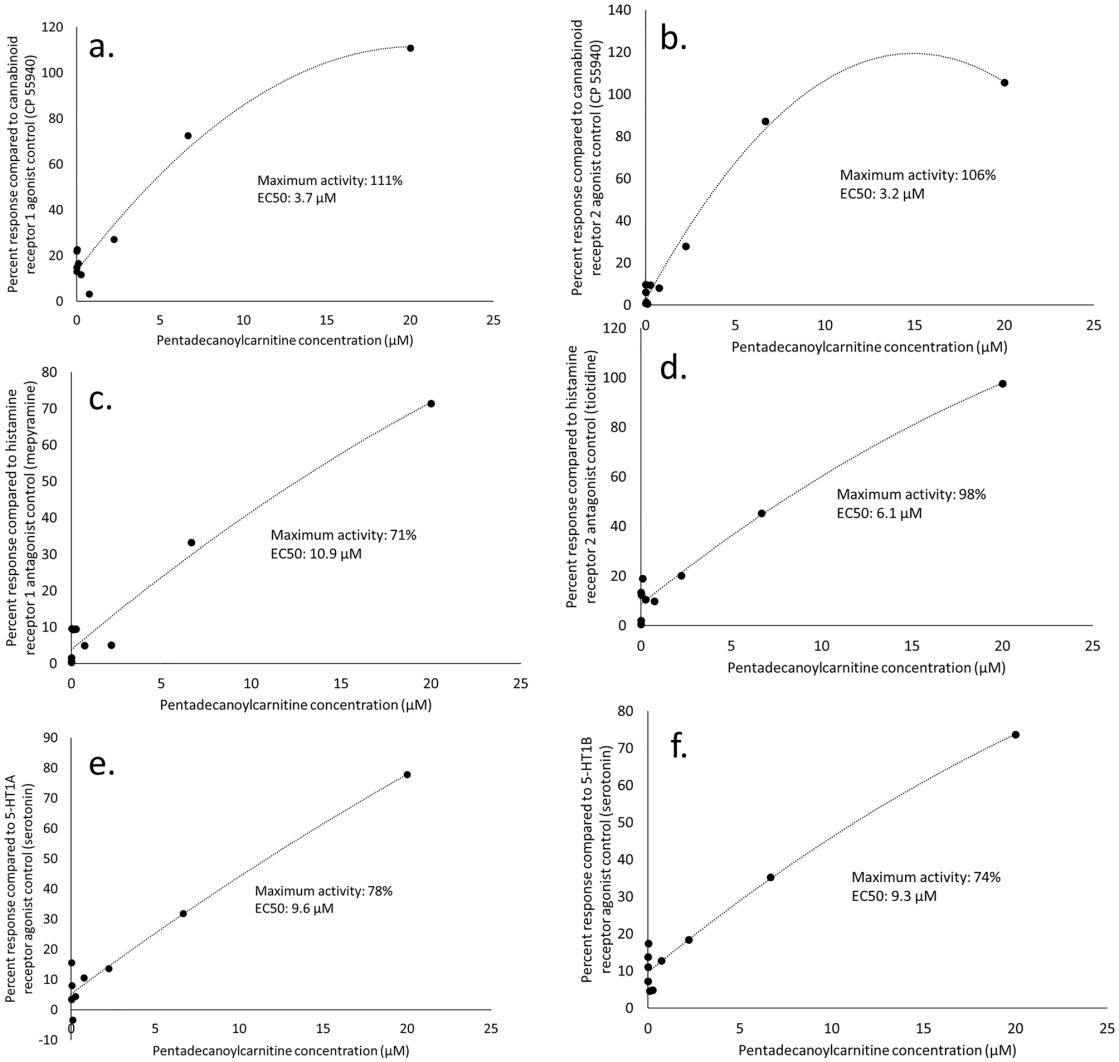
Targeted pharmacological dose–response activities of pentadecanoylcarnitine at ten concentrations (ranging from 0.1 to 20 µM), compared to internal positive controls. Detected pentadecanoylcarnitine activities included (**a**) cannabinoid receptor 1 agonist, (**b**) cannabinoid receptor 2 agonist, (**c**) histamine receptor 1 antagonist, (**d**) histamine receptor 2 antagonist, (**e**) 5-HT1A agonist, (**f**) 5-HT1B agonist.

Applying a gold standard, cell-based, G-protein coupled receptor cAMP modulation assay routinely used to identify compounds with cannabinoid receptor 1 and 2 (CB1 and CB2) agonist activities, pentadecanoylcarnitine had dose–response agonist activities for both CB1 and CB2, with half-maximal effective concentrations of 3.7 and 3.2 µM, respectively (Figs. 4a and b). Maximum pentadecanoylcarnitine CB1 and CB2 agonist activities (111% and 106%, respectively) were greater than the positive control (CP 55940). These data effectively demonstrated pentadecanoylcarnitine as a full CB1 and CB2 agonist.

Pentadecanoylcarnitine also had dose–response antagonist activities for both histamine H1 and H2 receptors, with half-maximal inhibitory concentrations of 10.9 and 6.1 µM, respectively (Figs. 4c and d). Maximum pentadecanoylcarnitine histamine H1 and H2 receptor antagonist activities were 71% and 98%, respectively, compared to the positive control (histamine), demonstrating its role as an antihistamine nutrient.

In addition, pentadecanoylcarnitine had dose–response agonist activities for both serotonin 1A and 1B (5-HT1A and 5-HT1B) receptors, with half-maximal effective concentrations of 9.6 and 9.3 µM, respectively (Figs. 4e and f). Maximum pentadecanoylcarnitine 5-HT1A and 5-HT1B agonist activities were 78% and 74%, respectively, compared to the positive control (serotonin hydrochloride), demonstrating its role as a serotonin mimic.

Effective concentrations of pentadecanoylcarnitine across all six pharmacological targets, ranging from 3.2 to 10.9 µM, were aligned with similar concentrations, ranging from 0.7 to 6.7 µM, that resulted in clinically relevant activities in human cell systems mimicking various disease states.

## Discussion

Here, we report pentadecanoylcarnitine, a long-chain C15:0 acylcarnitine, as an active and natural compound with clinically relevant and pleiotropic activities. As the name suggests, long-chain acylcarnitines are a product of a long-chain fatty acid combined with carnitine. This category of metabolites is made almost entirely within cells, and they enable β-oxidation-generated energy by mitochondria that, in turn, supports cellular activities^17^. Given this general understanding of long-chain acylcarnitines, it is expected that C15:0 combines intracellularly with carnitine to endogenously produce pentadecanoylcarnitine.

Relevant to mitochondrial support, we previously reported that C15:0 at concentrations between 10 and 50 µM restored mitochondrial function in human cell-based studies, including reduction of mitochondrial reactive oxygen species^2^. The current study offers further insights into how intracellular C15:0 may improve mitochondrial function, including enabling the generation of a long chain acylcarnitine, pentadecanoylcarnitine. Unexpectedly, beyond the role of supporting mitochondrial function, pentadecanoylcarnitine had potent pleiotropic activities, including directly attenuating disease in human cell-based systems and effectively targeting clinically relevant receptors.

The current study demonstrated clinically relevant and dose-dependent activities of pentadecanoylcarnitine at concentrations between 0.7 and 6.7 µM. Specifically, pentadecanoylcarnitine had broad anti-inflammatory activities, including lowering multiple pro-inflammatory cytokines and chemokines (including IL-1α and MCP-1) in four human cell systems mimicking chronic inflammation, autoimmune disease, lung inflammation, chronic obstructive pulmonary disease, dermatitis, psoriasis, cancer, and cardiovascular disease. These discovered activities of pentadecanoylcarnitine go against the general knowledge of long-chain acylcarnitines. Namely, higher circulating concentrations of long-chain acylcarnitines have traditionally been associated with a higher risk of age-related conditions, including inflammation, mitochondrial dysfunction, cardiovascular disease, type 2 diabetes, and osteoarthritis^18–21^. In the face of chronic disease states, raised concentrations of long-chain acylcarnitines have been attributed to dysfunctional mitochondria that spill long-chain acylcarnitines into serum and plasma instead of effectively using them for fuel^22^. Interestingly, odd-chain acylcarnitines decrease with age while circulating concentrations of long-chain acylcarnitines tended to increase with age^22^. Further studies are needed to evaluate how declining pentadecanoylcarnitine with age may contribute to age-related breakdown, including mitochondrial dysfunction.

The discovered broad and clinically relevant activities of pentadecanoylcarnitine in human cell-based systems were backed by its surprisingly effective, dose-dependent targeting of three key receptor families relevant to immune, metabolic, neurologic and mental health. Here, we showed that pentadecanoylcarnitine, an endogenous metabolite, activated CB1 and CB2 receptors in a dose–response fashion more effectively than the positive control, a synthetic form of tetrahydrocannabinol. Previously, only one endogenous cannabinoid was known to be a full agonist for both cannabinoid receptors: 2-arachidonoylglycerol^23^. Arachidonic acid, which is produced endogenously by the omega-6 essential fatty acid linoleic acid, serves as the lipid moiety for the two most studied endocannabinoids, 2-arachidonoylglycerol and anandamide^14,24^. In a similar fashion, we propose the essential odd-chain saturated fatty acid, pentadecanoic acid (C15:0), as the lipid moiety for a newly discovered, full-acting endocannabinoid, pentadecanoylcarnitine.

The endocannabinoid system (ECS) has gained increasing attention due to its ubiquitous role in supporting both physiological and mental health^25^. While current evidence supports that the ECS may be targeted to treat type 2 diabetes, Parkinson’s and Alzheimer’s diseases, pain, anxiety and stress, sleep disorders, inflammation and immune diseases, and cancer^26–32^, the very short half-lives of circulating 2-arachidonoylglycerol and anandamide have been barriers to using these two endocannabinoids as administered therapeutics^25^. The current study indicates that pentadecanoylcarnitine is a stable compound produced directly from exogenous C15:0, and clinical trials are warranted to assess the ability for pentadecanoylcarnitine to engage the ECS and provide therapeutic activities better than other endocannabinoids.

In addition to acting as a full CB1 and CB2 agonist, the current study showed that pentadecanoylcarnitine is a histamine 1 (H1R) and histamine 2 (H2R) receptor antagonist. Histamine receptor activation is a critical component of many allergic diseases, including asthma, dermatitis, and rhinitis, and compounds that inhibit histamine receptors are attractive therapeutic targets for these conditions^33^. In addition to inhibiting H1R and H2R, we found that pentadecanoylcarnitine lowered interferon-γ-inducible protein (IP-10) in a system mimicking Th1 type cutaneous inflammation. IP-10 is a chemokine that plays a role in chronic allergy inflammation, providing consistency between pentadecanoylcarnitine’s human cell-based efficacy and receptor-based activities^34^.

The global prevalence of allergies is on the rise, and with an estimated 1 in 5 people affected by asthma, allergic rhinitis, atopic dermatitis or food allergies, there is an urgent need to better understand underlying risk factors for this increase^35,36^. While there is a lack of studies evaluating associations between C15:0 and allergies, Stravik et al. evaluated relationships between maternal intake of cow’s milk during lactation with the onset of food allergies in offspring, including use of C15:0 as a biomarker of cow’s milk intake^37^. They found higher C15:0 dietary intake and resultant higher C15:0 concentrations in maternal red blood cells and breast milk and were associated with a lower risk of physician-diagnosed allergies in offspring at 12 months of age. While this study attributed cow’s milk intake during lactation as a potential means to reduce the risk of food allergies in infants, our current study suggests that a C15:0 metabolite, pentadecanoylcarnitine, could play a direct role in reducing the risk of allergies.

Our study also showed that pentadecanoylcarnitine is an endogenous 5-HT1A and 5-HT1B agonist. 5-HT receptors are present in both the brain and body, including 5-HT1A and 5-HT1B. 5-HT1A agonists, such as buspirone, are used as antidepressants and anxiolytics^38^. Other proposed therapeutic roles of 5-HT1A agonists include sexual enhancement, analgesia, L-DOPA dyskinesia with Parkinson’s disease, schizophrenia, and cognition support. 5-HT1B agonists can be used to manage depression and anxiety, in addition to reduction of aggression and impulsivity^38^. Further studies are needed to better understand how C15:0, through its acylcarnitine metabolite, pentadecanoylcarnitine, may naturally support mental health.

When bottlenose dolphins were fed a modified, higher-C15:0 fish diet, both C15:0 and pentadecanoylcarnitine serum concentrations increased within the first month; these concentrations remained higher than baseline controls throughout the 6-month study. This indicates that pentadecanoylcarnitine may be readily produced and maintained in the body by modifying exogenous intake of C15:0. Our assumption that dolphins’ pentadecanoylcarnitine serum concentrations increased secondarily to higher dietary intake of C15:0 is supported by the observed rise in pentadecanoylcarnitine’s importance over time, including becoming the highest ranked of all 819 measured metabolites after 6 months on the modified fish diet. While it is believed that pentadecanoylcarnitine is a direct metabolite of C15:0, pentadecanoylcarnitine concentrations were not measured in the modified fish diet; as such, it cannot be ruled out that the modified fish diet contained bioavailable pentadecanoylcarnitine that contributed to its higher serum concentrations.

Regardless of endogenous or exogenous source, this study demonstrated that dietary interventions can effectively increase long-term, circulating C15:0 and pentadecanoylcarnitine concentrations. We have previously reported that this modified diet study resulted in healthier, lower total cholesterol and insulin, as well as alleviated anemia, which was attributed to increased C15:0 concentrations^11^. Further studies are needed to evaluate the potential role of pentadecanoylcarnitine on these clinically relevant effects.

In summary, similar to other essential fatty acids, we have demonstrated that C15:0 itself, and now a C15:0 metabolite, have pleiotropic effects with expected broad health benefits. Specifically, pentadecanoylcarnitine has potent pro-endocannabinoid, serotonin-supporting, and antihistamine activities relevant to promoting both physical and mental health, including its ability to regulate inflammation, pain, mood, sleep, and stress. Due to population-wide decreases in whole fat milk intake^39^, paired with declining circulating C15:0 concentrations^40^, further studies are needed to evaluate possible links between the global rise in allergies, mental health conditions, and sleep disorders and C15:0 nutritional deficiencies.

## Methods

### Ethics declarations

The U.S. Navy Marine Mammal Program is accredited by the Association for Assessment and Accreditation of Laboratory Animal Care International and adheres to the national standards of the United States Public Health Service Policy on the Humane Care and Use of Laboratory Animals and the Animal Welfare Act. As required by the Department of Defense, the Program’s animal care and use program is routinely reviewed by the Naval Information Warfare Center Pacific (NIWC Pac) Institutional Animal Care and Use Committee and the Navy Bureau of Medicine and Surgery. This study was conducted under the NIWC Pac IACUC-approved animal care and use protocol #101–2012 (BUMED NRD-801). Reporting in this paper follows the recommendations in the ARRIVE guidelines.

### Modified diet study for dolphins

Methods used for this study have been described previously^11^. Briefly, the study population was from a managed population of adult bottlenose dolphins (*Tursiops truncatus*) living in San Diego Bay, California (32.6500°N, 117.1900°W) cared for by the U.S. Navy Marine Mammal Program (MMP). Dolphins placed on the modified diet (n = 20) and dolphins maintained on the baseline diet (n = 10) were matched by age and sex. The baseline diet was based on a typical Navy dolphin diet fed over the past decade (75% kcals capelin + 25% kcals herring/squid). The modified diet (50% kcals mullet + 25% kcals capelin + 25% kcals herring/squid) aimed to increase dietary odd-chain saturated fatty acid intake based upon the original pilot study^16^. Twenty dolphins were moved to the modified diet for six months. Ten dolphins, housed in the same environment, were maintained on the baseline diet to control for potential non-dietary environmental factors that may have occurred during the study. Previously reported sample collection protocols for MMP dolphins were used in this study^16^.

### Serum metabolomics

#### Sample preparation

Serum samples archived at − 80 °C were prepared using the automated MicroLab STAR® system (Hamilton Company). Proteins were precipitated with methanol under vigorous shaking for 2 min (Glen Mills GenoGrinder 2000) followed by centrifugation. The resulting extract was divided into five fractions: two for analysis by two separate reverse phase (RP)/UPLC-MS/MS methods with positive ion mode electrospray ionization (ESI), one for analysis by RP/UPLC-MS/MS with negative ion mode ESI, one for analysis by HILIC/UPLC-MS/MS with negative ion mode ESI, and one sample reserved for backup. Samples were placed briefly on a TurboVap® (Zymark) to remove the organic solvent. The sample extracts were stored overnight under nitrogen before preparation for analysis.

#### Quality assurance and control

Several types of controls were analyzed in concert with the experimental samples: a pooled matrix sample generated by taking a small volume of each experimental sample (or alternatively, use of a pool of well-characterized human plasma) served as a technical replicate throughout the data set; extracted water samples served as process blanks; and a cocktail of QC standards that were carefully chosen not to interfere with the measurement of endogenous compounds were spiked into every analyzed sample, allowed instrument performance monitoring and aided chromatographic alignment.

#### Bioinformatics

The informatics system consisted of four major components, the Laboratory Information Management System (LIMS), the data extraction and peak-identification software, data processing tools for QC and compound identification, and a collection of information interpretation and visualization tools for use by data analysts. The hardware and software foundations for these informatics components were the LAN backbone, and a database server running Oracle 10.2.0.1 Enterprise Edition. Peaks were quantified using area-under-the-curve. For studies spanning multiple days, a data normalization step was performed to correct variation resulting from instrument inter-day tuning differences. Essentially, each compound was corrected in run-day blocks by registering the medians to equal one (1.00) and normalizing each data point proportionately (termed the “block correction”). For studies that did not require more than one day of analysis, no normalization is necessary, other than for purposes of data visualization. In certain instances, biochemical data may have been normalized to an additional factor (e.g., cell counts, total protein as determined by Bradford assay, osmolality) to account for differences in metabolite levels due to differences in the amount of material present in each sample. Two-way ANOVA of main effects models, including study group, month, and sex, were used to determine primary drivers of differences in the metabolome.

#### Principal components analysis and hierarchical clustering

Each principal component was a linear combination of every metabolite and the principal components were uncorrelated. The number of principal components was equal to the number of observations. The first principal component was computed by determining the coefficients of the metabolites that maximized the variance of the linear combination. The second component found the coefficients that maximize the variance with the condition that the second component was orthogonal to the first. The third component was orthogonal to the first two components and so on. The total variance was defined as the sum of the variances of the predicted values of each component (the variance is the square of the standard deviation), and for each component, the proportion of the total variance was computed. Hierarchical clustering was used as an unsupervised method for clustering the data to show any large-scale differences. Complete clustering using the Euclidean distance was applied, where each sample was a vector with all metabolite values.

#### Random forest regression

Random forest, a supervised classification technique based on an ensemble of decision trees, was used to provide “importance” rank ordering of serum biochemicals that changed due to the modified diet. A random subset of the data with identifying true class information was selected to build the tree (“bootstrap sample” or “training set”), and then the remaining data, the “out-of-bag” (OOB) variables, were passed down the tree to obtain a class prediction for each sample. This process was repeated thousands of times to produce the forest. The final classification of each sample was determined by computing the class prediction frequency (“votes”) for the OOB variables over the whole forest. This method was unbiased since the prediction for each sample was based on trees built from a subset of samples that did not include that sample. When the full forest was grown, the class predictions were compared to the true classes, generating the “OOB error rate” as a measure of prediction accuracy. Thus, the prediction accuracy was an unbiased estimate of how well one can predict sample class in a new data set. To determine which biochemicals made the largest contribution to the classification, a “variable importance” measure was computed. The “Mean Decrease Accuracy” (MDA) was used as this metric. The MDA was determined by randomly permuting a variable, running the observed values through the trees, and then reassessing the prediction accuracy.

### Pentadecanoylcarnitine synthesis

**Figure 5:**
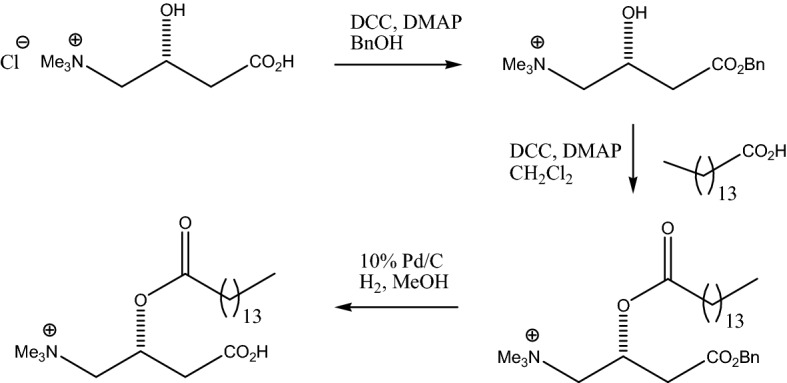
Synthesis of pentadecanoylcarnitine using a 3-step process.

**Figure 6:**
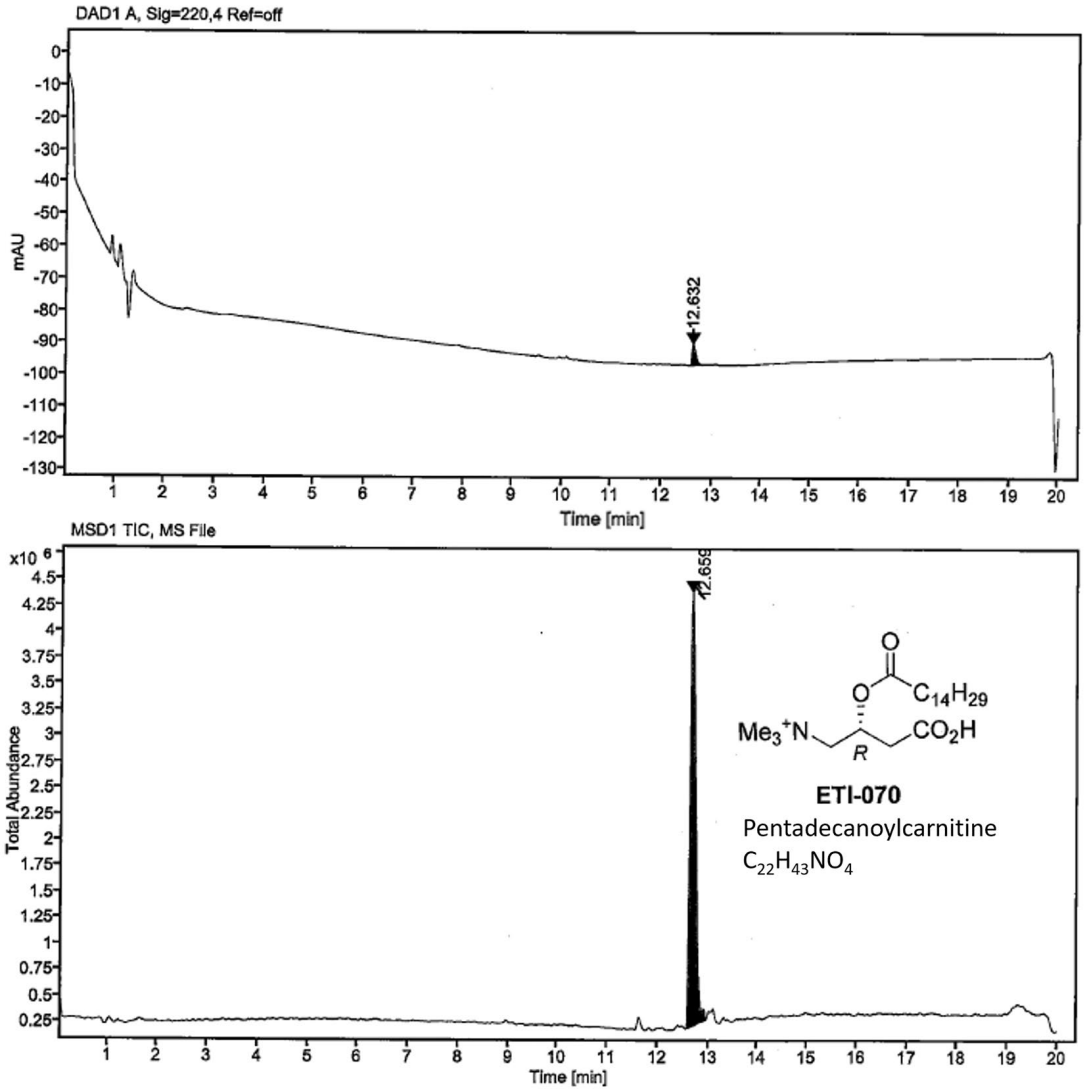
Liquid chromatography-mass spectrometry of synthesized pentadecanoylcarnitine.

Given the high ranking of pentadecanoylcarnitine from the modified fish diet study with dolphins, and its likelihood as a C15:0 metabolite, this compound was synthesized using a 3-step process described below (Fig. 5). The final product was confirmed as pentadecanoylcarnitine using liquid chromatography mass spectrometry (Fig. 6).

A suspension of 1.0 g of L-carnitine hydrochloride, 1.1 g of dicyclohexylcarbodiimide (DCC), and 60 mg of dimethylaminopyridine (DMAP) were stirred in 10 ml of benzyl alcohol at room temperature for 40 h. The resulting heterogeneous mixture was filtered through Celite, washing the solids with CH_2_Cl_2_. The filtrate was concentrated on a rotovap. The residue was then chromatographed on silica gel (20 g) eluting with CH_2_Cl_2_ / MeOH gradient (5–20%) to afford 0.86 g white solid.

L-carnitine benzyl ester (288 mg), pentadecanoic acid (240 mg), DMAP (12 mg), and DCC (227 mg) were stirred in 5 ml of CH_2_Cl_2_ at room temperature for 48 h. The resulting mixture was filtered through Celite and the solids washed with CH_2_Cl_2_. The filtrate was concentrated and the residue was chromatographed on silica gel (10 g) eluting with CH_2_Cl_2_ / MeOH gradient (0–8%) to yield 110 mg of product.

Pentadecanoylcarnitine benzyl ester (110 mg) and 10 mg of 10% Pd/C catalyst were added to 5 ml of MeOH. A hydrogen balloon was attached to the flask and after several vacuum purges, the reaction was stirred at room temperature for 24 h. The mixture was filtered through Celite, rinsing the solids with MeOH. The filtrate was concentrated to a white solid (90 mg).

### Cell exposure studies

Human primary cells in Eurofins’ BioMAP systems were used at early passage (passage 4 or earlier) to minimize adaptation to cell culture conditions and preserve physiological signaling responses. All cells were from a pool of multiple donors (n = 2 to 6), commercially purchased and handled according to the recommendations of the manufacturers. Human blood derived CD14 + monocytes were differentiated into macrophages in vitro before being added to the *l*Mphg system.

Cell types and stimuli used in each system were as follows: 3C system [HUVEC + *(IL-1β, TNFα and IFNγ)*], 4H system [HUVEC + *(IL-4 and histamine)*], LPS system [PBMC and HUVEC + *LPS (TLR4 ligand)*], SAg system [PBMC and HUVEC + *TCR ligands*], BT system [CD19 + B cells and PBMC + *(α-IgM and TCR ligands)*], BF4T system [bronchial epithelial cells and HDFn + *(TNFα and IL-4)*], BE3C system [bronchial epithelial cells + *(IL-1β, TNFα and IFNγ)*], CASM3C system [coronary artery smooth muscle cells + *(IL-1β, TNFα and IFNγ)*], HDF3CGF system [HDFn + *(IL-1β, TNFα, IFNγ, EGF, bFGF and PDGF-BB)*], KF3CT system [keratinocytes and HDFn + *(IL-1β, TNFα, IFNγ and TGFβ)*], MyoF system [differentiated lung myofibroblasts + *(TNFα and TGFβ)*] and Mphg system [HUVEC and M1 macrophages + *Zymosan (TLR2 ligand)*].

To evaluate dose response, three concentrations of pentadecanoylcarnitine (0.7, 2.2, and 6.4 µM) were tested in each of the 12 cell systems. The doses were chosen given the known circulating concentrations of the parent compound, pentadecanoic acid (10 μM) and an anticipated metabolite level of no more than approximately 50% (5 μM); known concentrations of a closely related acylcarnitine, palmitoylcarnitine (1 uM); known concentrations of the only other full acting endocannabinoid, anandamide (3–8 μM), which is also an essential fatty acid metabolite.

Systems were derived from either single cell types or co-culture systems. Adherent cell types were cultured in 96 or 384-well plates until confluence, followed by the addition of PBMC (SAg and LPS systems). The BT system consisted of CD19 + B cells co-cultured with PBMC and stimulated with a BCR activator and low levels of TCR stimulation. Test agents prepared in either DMSO (small molecules; final concentration ≤ 0.1%) or PBS (biologics) were added at the indicated concentrations 1-h before stimulation and remained in culture for 24 h or as otherwise indicated (48 h, MyoF system; 72 h, BT system (soluble readouts); 168 h, BT system (secreted IgG)). Each plate contained drug controls (e.g., legacy control test agent colchicine at 1.1 μM), negative controls (e.g., non-stimulated conditions) and vehicle controls (e.g., 0.1% DMSO) appropriate for each system.

Direct ELISA was used to measure biomarker levels of cell-associated and cell membrane targets. Soluble factors from supernatants were quantified using either HTRF® detection, bead-based multiplex immunoassay or capture ELISA. Overt adverse effects of test agents on cell proliferation and viability (cytotoxicity) were detected by sulforhodamine B (SRB) staining, for adherent cells, and alamarBlue® reduction for cells in suspension. For proliferation assays, individual cell types were cultured at subconfluence and measured at time points optimized for each system (48 h: 3C and CASM3C systems; 72 h: BT and HDF3CGF systems; 96 h: SAg system). Cytotoxicity for adherent cells was measured by SRB (24 h: 3C, 4H, LPS, SAg, BF4T, BE3C, CASM3C, HDF3CGF, KF3CT, and Mphg systems; 48 h: MyoF system), and by alamarBlue staining for cells in suspension (24 h: SAg system; 42 h: BT system) at the time points indicated.

#### Profile analysis

Biomarker activities were annotated when 2 or more consecutive concentrations changed in the same direction relative to vehicle controls, were outside of the significance envelope and had at least one concentration with an effect size > 20% (|log_10_ *ratio*|> 0.1). Biomarker key activities were described as modulated if these activities increased in some systems but decreased in others. Cytotoxic conditions were noted when total protein levels decreased by more than 50% (log_10_ *ratio* of SRB or alamarBlue levels < -0.3) and were indicated by a thin black arrow above the X-axis. A compound was considered to have broad cytotoxicity when cytotoxicity was detected in 3 or more systems. Concentrations of test agents with detectable broad cytotoxicity were excluded from biomarker activity annotation and downstream benchmarking, similarity search and cluster analysis. Antiproliferative effects were defined by an SRB or alamarBlue log10 *ratio* value < − 0.1 from cells plated at a lower density and were indicated by grey arrows above the X-axis. Cytotoxicity and antiproliferative arrows only required one concentration to meet the indicated threshold for profile annotation.

### Clinically relevant activities

It was hypothesized that pentadecanoylcarnitine would have clinically relevant, receptor-based activities. To test this hypothesis, the Eurofins/DiscoverX SafetyScan47® panel was used to test C15:0 at 10 concentrations for agonist and antagonist activities across 78 assays and 47 genes and targets that are relevant to mechanisms of action, including G-protein coupled receptors, kinases, transporters, ion channels, nuclear receptors, and non-kinase enzymes. Detailed methods used for these assays and assay readouts are available from Eurofins/DiscoverX.

## Data Availability

The datasets generated during and/or analyzed as part of the current study are available from the corresponding author on reasonable request.
